# Analysis of gp41 epitopes in model viral membranes to study HIV-1 neutralization

**DOI:** 10.1186/1742-4690-9-S2-P105

**Published:** 2012-09-13

**Authors:** T Reichart, M Baksh, M Zwick, M Finn, P Dawson

**Affiliations:** 1 The Scripps Research Institute, La Jolla, CA, USA

## Background

The membrane proximal external region (MPER) of the HIV surface glycoprotein gp41 is the binding site for several potent broadly neutralizing antibodies. These antibodies recognize highly conserved linear peptide epitopes and antigens derived from this region have the potential to be important components of a vaccine directed against HIV-1. Highly conserved peptide epitopes for these antibodies are sterically obscured from the immune system by an intimate association with the viral membrane and by the presence of a large trimeric glycoprotein spike. As a result, previous attempts to immunize with linear peptides have failed to elicit broadly neutralizing antibodies.

## Methods

In order to better mimic the structural features of the MPER domain with an eye towards better understanding of epitope-antibody interactions near membranes, peptide mimics derived from HIV gp41 have been synthesized with a variety of structural changes including positioning of the epitope with respect to the membrane as well as the presence and nature of an anchoring transmembrane domain. These peptide mimics have been incorporated into membrane bilayer mimics called “Nanodiscs,” which are ~10 nm-diameter structures composed of a phospholipid bilayer ringed by an apolipoprotein-derived scaffold protein.

## Results

We have examined the affinity of HIV-1 neutralizing antibodies to these membrane-bound peptides to better understand the binding of these neutralizing antibodies to epitopes on or near a membrane. Significant differences in binding have been observed between peptides with different transmembrane domains.

## Conclusion

We have chemically synthesized peptide mimics of gp41 and incorporated them into artificial membrane bilayers. The peptides are anchored with both native and artificial transmembrane domains, and both the presence and nature of the transmembrane domain modulates antibody binding. Membrane presentation of peptide antigens with an appropriate transmembrane domain may be an important feature of vaccine design.

